# A prospective short-term study to evaluate methodologies for the assessment of disease extent, impact, and wound evolution in patients with dystrophic epidermolysis bullosa

**DOI:** 10.1186/s13023-022-02461-z

**Published:** 2022-08-13

**Authors:** Amy S. Paller, Elena Pope, Dan Rudin, Anna Malyala, Deborah Ramsdell, Ramsey Johnson, Hal Landy, Dedee F. Murrell, the DEB Investigators

**Affiliations:** 1 Departments of Dermatology and Pediatrics, Northwestern University Feinberg School of Medicine, 676 North St Clair Street, Suite 1600, Chicago, IL 60611-2997 USA; 2 Section of Pediatric Dermatology, The Hospital for Sick Children, and University of Toronto, Toronto, ON Canada; 3 Phoenix Tissue Repair, Boston, MA USA; 4 Department of Dermatology, St George Hospital, University of NSW, Sydney, NSW Australia

**Keywords:** Dystrophic epidermolysis bullosa, Clinician-assessed outcomes, Quality of life, Patient-reported outcomes, Disease severity, Outcome measures, EBDASI, iscorEB

## Abstract

**Background:**

Standardized assessments for dystrophic epidermolysis bullosa (DEB) are needed. This prospective, multicenter, 4-week, observational study was designed to evaluate DEB assessments for suitability as clinical trial endpoints.

**Methods:**

Patients with confirmed DEB diagnosis and ≥ 5 measurable wounds were included. The primary outcome was change from baseline in wound surface area (WSA) of 5 selected wounds by 3-dimensional imaging. Secondary endpoints were change from baseline in clinician global assessment (CGA) of WSA, wound characteristics, disease-related questionnaires and instruments (disease severity, quality of life [QoL], pain and disability, and itch), and tolerability of procedures.

**Results:**

Of 30 enrolled patients, 29 completed the study (of whom, 28 had recessive DEB). Median age was 17.8 years (range, 3.8–58.7). All patients developed new or recurrent wounds during the 4-week study. Of the wounds selected at baseline, 45/150 (30.0%) healed by week 2; an additional 38 healed by week 4, while 8 of those healed at week 2 had recurred by week 4 for a total of 75/150 (50.0%) healed wounds at week 4. Mean values for WSA, CGA, and disease-related questionnaire and instrument scores remained steady during this 4-week observational study. Of the 10 disease-related questionnaires and instruments assessed, the scores for the Epidermolysis Bullosa Disease Activity and Scarring Index (EBDASI) and the Instrument for Scoring Clinical Outcomes for Research of Epidermolysis Bullosa (iscorEB) did not substantially overlap between moderate and severe disease. Between mild and moderate disease, only the EBDASI scores did not substantially overlap.

**Conclusions:**

These results stress the dynamic nature of wounds, even during a 4-week period of observation, and suggest that a combination of clinician-assessed outcomes and patient-/caregiver-reported outcomes is needed to provide a comprehensive assessment of DEB severity and impact. In addition, these results support the use of EBDASI and iscorEB to monitor disease severity as both produced scores that did not substantially overlap between disease severity strata.

*Clinical trial registration* ClinicalTrials.gov, NCT02178969. Registered 4 June 2014, https://clinicaltrials.gov/ct2/show/NCT02178969.

**Supplementary Information:**

The online version contains supplementary material available at 10.1186/s13023-022-02461-z.

## Background

Epidermolysis bullosa (EB) encompasses a group of molecularly diverse diseases characterized by the development of blisters after minor mechanical trauma to the skin [1]. Blisters can be chronic (present for ≥ 12 weeks), recurrent (heal and re-blister in same location during a 12-week period), or resolved (completely heal in ≤ 12 weeks) [2]. There are 4 major types of inherited EB: EB simplex, junctional EB, dystrophic EB (DEB), and Kindler syndrome [3]. DEB is caused by mutations in the *COL7A1* gene, which encodes the alpha-chain of type VII collagen (C7) [4, 5], a protein essential for the formation of the fibrils that anchor the basement membrane to the underlying dermis [6]. DEB is inherited as either a dominant or recessive form. The recessive form typically presents with a more severe phenotype [7]; however, clinical presentation is highly variable and correlation between genotype and phenotype is difficult to establish [8].

DEB wounds are dynamic and occur due to the skin’s impaired resistance to external shear forces. This characteristic leads to a continued risk for mechanical injury, as well as impaired healing because of C7 deficiency or dysfunction. Characteristic features of DEB include lifelong skin fragility and healing with scarring. Repeated cycles of wound formation with healing and scarring lead to secondary signs, including joint contractures, mutilating deformities of hands and feet, malnutrition, growth retardation, and a highly increased risk of aggressive cutaneous squamous cell carcinoma [9, 10].

Management of DEB focuses on supportive care, with bandaging and wound infection prophylaxis the primary focus. Although recommendations for wound care have been published [11–16], none are uniformly accepted or implemented as standard [10]. Similarly, guidelines vary for early identification of squamous cell carcinoma, and management of pain, itch, and management of DEB sequelae, such as pseudosyndactyly, esophageal stenosis, feeding difficulties, and anemia.

Research conducted after this study ended has focused on the relationship between wound size and pain, itch, and wound chronicity [17, 18]. However, there are no recent comprehensive, longitudinal, multicenter studies assessing tools for DEB severity, impact on patients, or evolution. Longitudinal and comparative evaluations are needed to identify relevant outcome parameters for treatment studies in DEB. To address this need, this observational study evaluated various tools for EB assessment.

## Results

### Patient disposition, baseline characteristics, and wound selection/characteristics

**Fig. 1:**
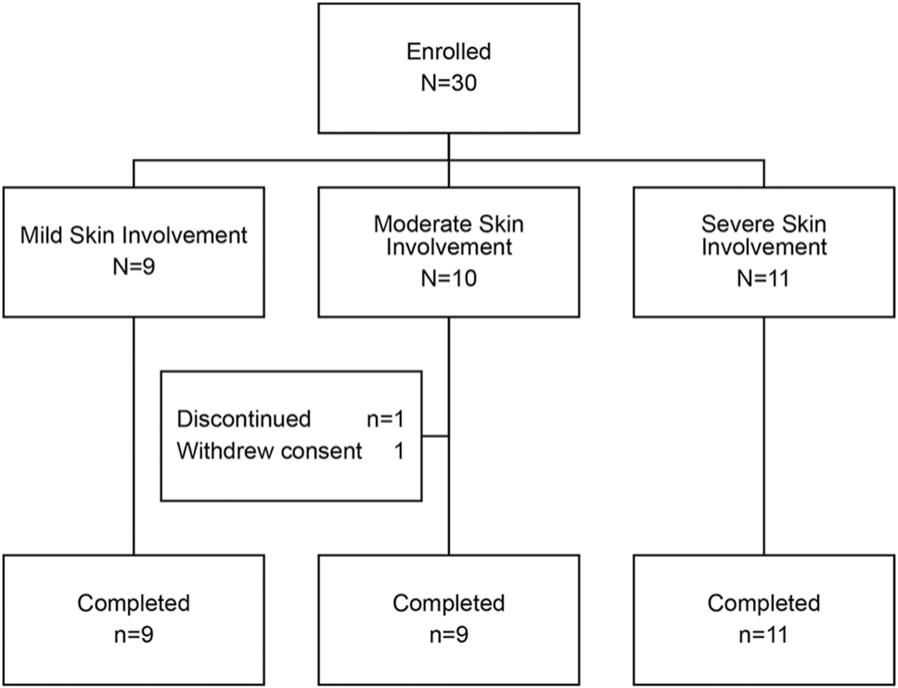
Patient disposition

A total of 30 patients (15 adult and 15 pediatric patients) with DEB were enrolled at 9 centers across 8 countries (Additional file 1); 29 patients completed the study (Fig. 1). One patient had dominant DEB; the remaining 29 patients (of whom, 28 completed the study) had a recessive DEB (Table 1). Based on a combination of the extent of BSA affected by wounds, DEB subtype, body parts affected, and presence of non-cutaneous symptoms, 9 patients were rated by the investigator to have mild DEB, 10 patients moderate DEB, and 11 patients severe DEB (Table 1). The median age was 17.8 years (range, 3.8–58.7 years; Table 1). Fourteen patients had a history of pseudosyndactyly (2–10 years, 4 patients; 10–18 years, 4 patients; > 18 years, 6 patients).

**Table 1:** Demographic and baseline clinical characteristics

	Mild N = 9	Moderate N = 10	Severe N = 11	Overall N = 30
Age (years)				
Mean (SD)	18.3 (18.1)	29.1 (17.9)	19.1 (10.9)	22.2 (16.0)
Median (range)	9.1 (3.9–58.7)	33.2 (3.8–50.7)	15.9 (4.1–37.9)	17.8 (3.8–58.7)
Age group, n (%)				
≤ 2 years	0	0	0	0
> 2 to 10 years	5	3	2	10 (33.3)
> 10 to 18 years	1	0	4	5 (16.7)
> 18 years	3	7	5	15 (50.0)
Sex. n (%)				
Male	7	3	5	15 (50.0)
Female	2	7	6	15 (50.0)
Race, n (%)				
White	9	7	10	26 (86.7)
Asian	0	2	0	2 (6.7)
Other	0	1	1	2 (6.7)
BMI category (patients < 18 years), n (%)				
Underweight (< 5th percentile)	1	3	3	7 (46.7)
Healthy weight (5th to 75th percentile)	5	0	3	8 (53)
Overweight (> 75th percentile)	0	0	0	0
BMI category (patients ≥ 18 years), n (%)				
Underweight (16 to < 18.5 kg/m^2^)	0	3	4	7 (46.7)
Healthy weight (18.5 to < 25.0 kg/m^2^)	2	4	1	7 (46.7)
Overweight (≥ 25 kg/m^2^)	1	0	0	1 (6.7)
Primary DEB diagnosis and subtype, n (%)				
Recessive DEB	8	10	11	29 (96.7)
Generalized severe	0	0	9	9 (30.0)
Generalized intermediate	6	9	2	17 (56.7)
Inversa	2	1	0	3 (10.0)
Dominant DEB	1	0	0	1 (3.3)
Other subtype	1	0	0	1 (3.3)
Medical history, n (%)				
Dysphagia	5	7	11	23 (76.7)
Esophageal stenosis	3	7	10	20 (66.7)
Microstomia	3	3	7	13 (43.3)
Squamous cell carcinoma^a^	0	2	1	3 (10.0)
Surgical/procedural history, n (%)				
Esophageal dilation	0	4	5	9 (30.0)
Hand repair operation	0	3	3	6 (20.0)
Gastrostomy	0	2	2	4 (13.3)
Finger repair operation	0	1	2	3 (10.0)
Gastrointestinal tube insertion	0	0	3	3 (10.0)

*BMI* body mass index, *DDEB* dominant dystrophic epidermolysis bullosa, *DEB* dystrophic epidermolysis bullosa

^a^All in patients aged > 37 years

Patients chose target wounds that had the greatest impact on their life quality. Reasons cited included: pain (40/90, 44.4%), interference with daily activities (35/90, 38.9%), chronicity (30/90, 33.3%), and pruritus (30/90, 33.3%). Only 3/90 (3.3%) target wounds were selected by patients/caregivers because they were large. The most common reason clinicians selected target wounds for imaging and observation was recent onset (38/112, 33.9%); only 8/112 (13.3%) target wounds were selected by the clinician because of large size.

**Fig. 2:**
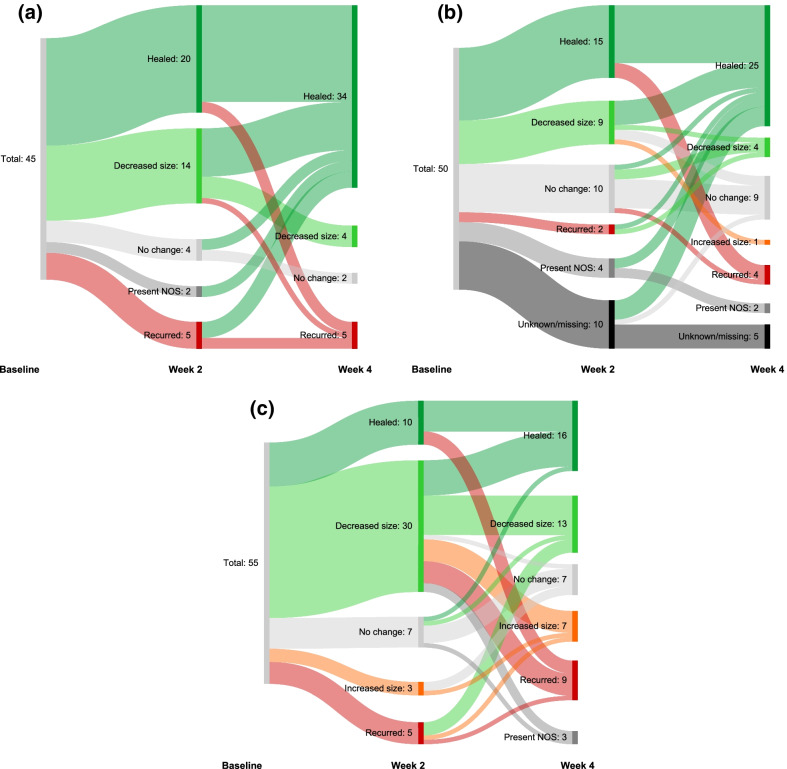
Target wound outcomes for patients with **A** mild, **B** moderate, or **C** severe disease. *NOS* not otherwise specified

All 30 patients developed new or recurrent wounds during the 4-week observation period. The median (range) number of new or recurrent wounds were: 14.0 (8.0–28.0), 9.5 (3.0–24.0), and 14.0 (3.0–28.0) for mild, moderate, and severe disease, respectively. Overall, 45/150 (30.0%) selected wounds were healed at week 2; an additional 38 healed by week 4, while 8 of those healed at week 2 had recurred by week 4 for a total of 75/150 (50.0%) healed wounds at week 4 (Fig. 2). Of the 5 wounds selected for each patient, the mean (SD) baseline size of wounds that healed at week 2 was 2.8 cm^2^ (4.7) and at week 4 was 3.5 cm^2^ (4.8). The baseline sizes of wounds that had recurred at week 2 and week 4 were 4.1 cm^2^ (4.3) and 9.1 cm^2^ (9.8), respectively. The baseline sizes of wounds that persisted at week 2 and week 4 were 18.4 cm^2^ (43.8) and 24.7 cm^2^ (54.2), respectively. Of 39 wounds that were > 5 cm^2^ at baseline, 6 (15.8%) healed and 1 (2.6%) recurred by week 2; 6 (15.4%) each had healed or recurred by week 4. Greater proportions of wounds that were healed at week 2 or week 4 were uninfected at baseline and had more epithelization, healthy granulation tissue, no odor, red peri-wound tissue, and no-to-scant exudate that was clear/not purulent compared with wounds that persisted or recurred. There were no obvious trends for wound depth or other baseline parameters (Additional file 2).

### Quantitative measurements of wound surface area

Mean (SD) WSA (body surface area covered by selected wounds) was 11.6% (20.7%) at baseline, 7.1% (14.2%) at week 2, and 5.9% (13.1%) at week 4. Most (83.1%) of the 544 wounds were ≤ 10 cm^2^ (Table 2). Values calculated from remote assessment of 2-dimensional digital photographs were consistently lower than direct visualization for patients with moderate or severe disease (Table 3). The mean percentage of BSA affected by all wounds was < 15% throughout the study, even in patients with severe disease (mean, 13.1%) (Table 3). CGA of WSA as a percentage of BSA changed little during the 4-week period of observation; from baseline to week 4 mean changes (SD) were − 0.2 cm^2^ (4.0) by direct visualization and − 0.1 cm^2^ (2.9) by remote assessment (Table 3).

**Table 2:** Wound surface area of selected wounds by 3-dimensional quantitative imaging

	Mild N = 9	Moderate N = 10	Severe N = 11	Overall N = 30
Number of wounds	165	174	205	544
WSA (cm^2^)				
Mean (SD)	1.4 (3.2)	5.0 (12.3)	15.6 (44.9)	7.9 (29.1)
Median (range)	0.4 (0.0–‍22.9)	1.1 (0.0–‍82.2)	5.5 (0.0–‍371.2)	1.1 (0.0–‍371.2)
WSA category, n (%)				
≤ 10 cm^2^	160 (97.0)	157 (90.2)	135 (65.9)	452 (83.1)
10 to ≤ 30 cm^2^	5 (3.0)	10 (5.7)	55 (26.8)	70 (12.9)
> 30 cm^2^	0	7 (4.0)	15 (7.3)	22 (4.0)

*WSA* wound surface area

**Table 3:** Clinician global assessment of wound surface area as percentage of body surface area by in-person direct visualization and remote (photographic) assessment of 2-dimensional digital images

	CGA of WSA (% of BSA)											
	Mild N = 9			Moderate N = 10			Severe N = 11			Overall N = 30		
	Direct	Remote	Difference	Direct	Remote	Difference	Direct	Remote	Difference	Direct	Remote	Difference
Baseline	n = 9			n = 10			n = 11			n = 30		
Mean (SD)	2.8 (2.0)	2.5 (2.2)	0.3 (0.6)	8.4 (7.7)	6.4 (6.4)	2.0 (3.9)	13.1 (9.0)	9.3 (6.3)	3.8 (4.3)	8.4 (8.1)	6.3 (5.9)	2.1 (3.7)
Median (range)	2.1 (0.2–‍6.0)	2.2 (0.1–‍6.5)	0.1 (− 0.5 to ‍1.3)	5.1 (0.4–‍20.7)	4.6 (0.4–‍20.5)	1.0 (− 0.2 to ‍13.0)	9.3 (3.8–‍32.0)	6.1 (4.1–‍23.7)	2.1 (− 0.3 to ‍12.1)	6.1 (0.2–‍32.0)	4.8 (0.1–‍23.7)	0.8 (− 0.5 to ‍13.0)
Week 2	n = 9			n = 9			n = 11			n = 29		
Mean (SD)	2.2 (1.5)	1.9 (1.6)	0.3 (0.5)	9.1 (8.9)	6.5 (5.7)	2.9 (7.3)	13.0 (9.4)	10.1 (7.2)	2.9 (4.8)	8.4 (8.7)	6.3 (6.3)	2.1 (5.0)
Median (range)	1.6 (0.1–‍4.8)	1.4 (0.2–‍4.8)	0.2 (− 0.4 to ‍1.1)	6.8 (1.0–‍27.2)	5.2 (0.8–‍19.0)	0.2 (− 0.8 to ‍22.0)	9.5 (5.4–‍31.3)	8.2 (3.4–‍27.6)	1.3 (− 1.8 to ‍14.7)	5.8 (0.1–‍31.3)	4.8 (0.2–‍27.6)	0.5 (− 1.8 to ‍22.0)
Week 4	n = 9			n = 9			n = 11			n = 29		
Mean (SD)	2.1 (1.7)	1.9 (2.0)	0.1 (0.6)	9.3 (10.1)	7.2 (8.4)	2.1 (7.4)	12.8 (10.2)	9.3 (7.8)	3.5 (4.4)	8.4 (9.4)	6.4 (7.3)	2.0 (4.9)
Median (range)	1.7 (0.2–‍5.4)	1.4 (0.1–‍5.7)	0.1 (− 0.7 to ‍1.4)	5.8 (0.7–‍26.7)	3.9 (0.5–‍26.4)	0.3 (− 3.7 to ‍21.0)	8.8 (4.9–‍33.4)	5.5 (2.3–‍29.0)	3.0 (− 0.6 to ‍15.2)	5.2 (0.2–‍33.4)	4.5 (0.1–‍29.0)	0.3 (− 3.7 to ‍21.0)
Change from baseline at week 4	n = 9			n = 9			n = 11			n = 29		
Mean (SD)	− 0.7 (1.1)	− 0.6 (1.1)	NA	0.3 (3.8)	0.3 (3.0)	NA	− 0.3 (5.5)	1.0 (4.0)	NA	− 0.2 (4.0)	− 0.1 (2.9)	NA
Median (range)	− 0.5 (− 3.0 to ‍0.5)	− 0.5 (− 2.3 to ‍0.8)		1.1 (− 5.8 to ‍6.0)	0.1 (− 3.5 to ‍5.9)		− 0.5 (− 9.9 to ‍7.6)	0.1 (− 8.9 to ‍5.4)		0.0 (− 9.9 to ‍7.6)	0.0 (− 8.9 to ‍5.9)	

CGAs for each method were calculated using the rule of nines

*BSA* body surface area, *CGA* clinician global assessment, *NA* not applicable, *WSA* wound surface area

### Impact of dystrophic epidermolysis bullosa

Based on QOLEB severity strata [19], most patients had moderate or severe impairment in QoL at baseline (Additional file 3). The QOLEB items with the greatest impact were involvement in sports, financial impact of family, ability to bathe or shower, ability to eat, and physical pain. Based on DLQI and CDLQI severity strata [20, 21], most patients with mild disease had small or moderate effect on QoL, while most patients with severe disease had moderate or very large effect on QoL (Additional file 3). The DLQI/CDLQI items with the greatest impact were symptoms/feelings (on both DLQI and CDLQI) and leisure (DLQI only). Mean HAQ/CHAQ disability indices were approximately 1 (out of 3) for patients with mild or moderate disease, and approximately 2 for patients with severe disease, indicating moderate-to-severe disability and severe-to-very severe disability, respectively [22].

### Outcome measures

#### Change over time

While EBDASI activity, damage, and total scores were generally stable during the 4-week observation period with small mean (SD) changes (Table 4), 2 patients experienced clinically meaningful improvement by week 4 (≥ 9-point reduction) and 11 patients experienced clinically meaningful deterioration (≥ 3-point increase) per the validated minimal clinically important differences of EBDASI total scores [23], thereby demonstrating the variable nature of this disease over the short-term without intervention. Overall, mean iscorEB scores (clinician, patient, and total scores) were stable (Table 4).

**Table 4:**
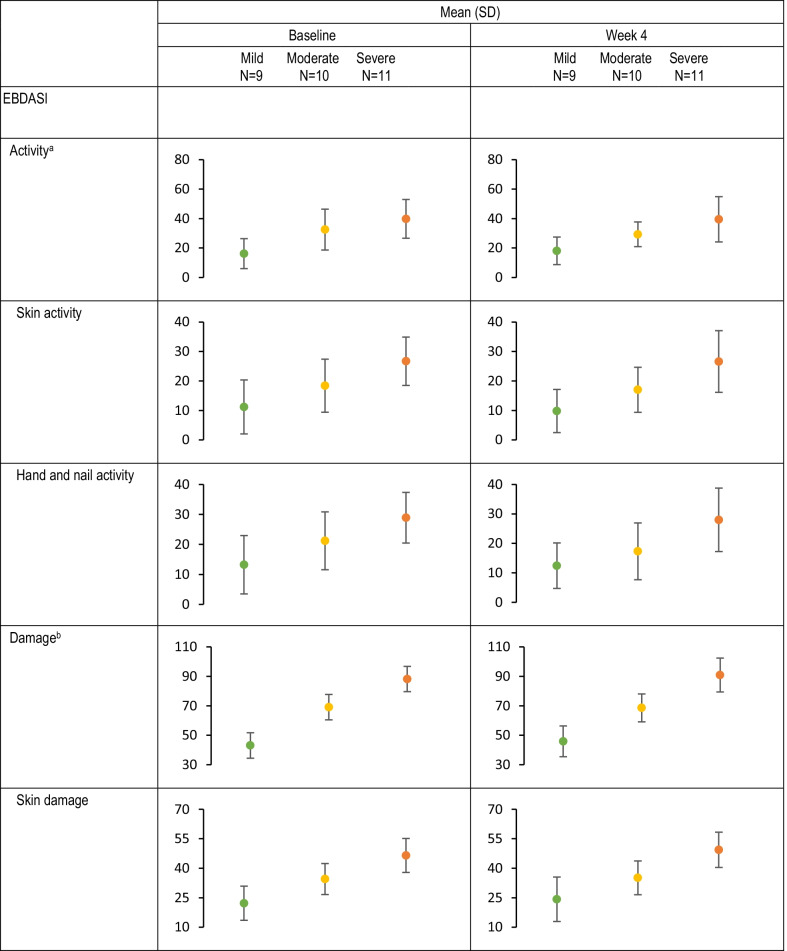

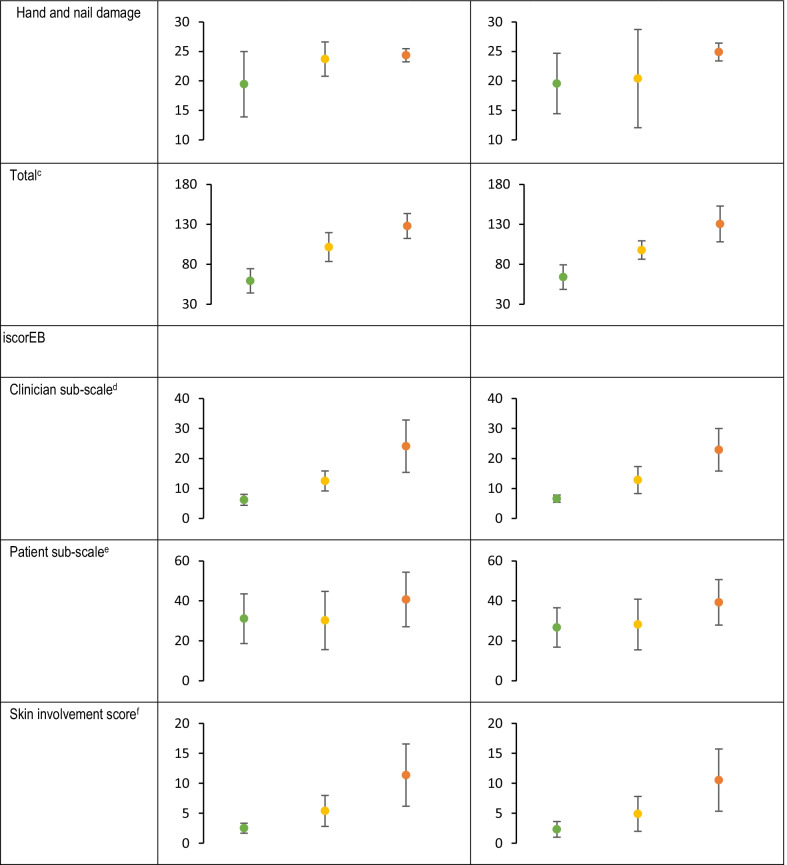

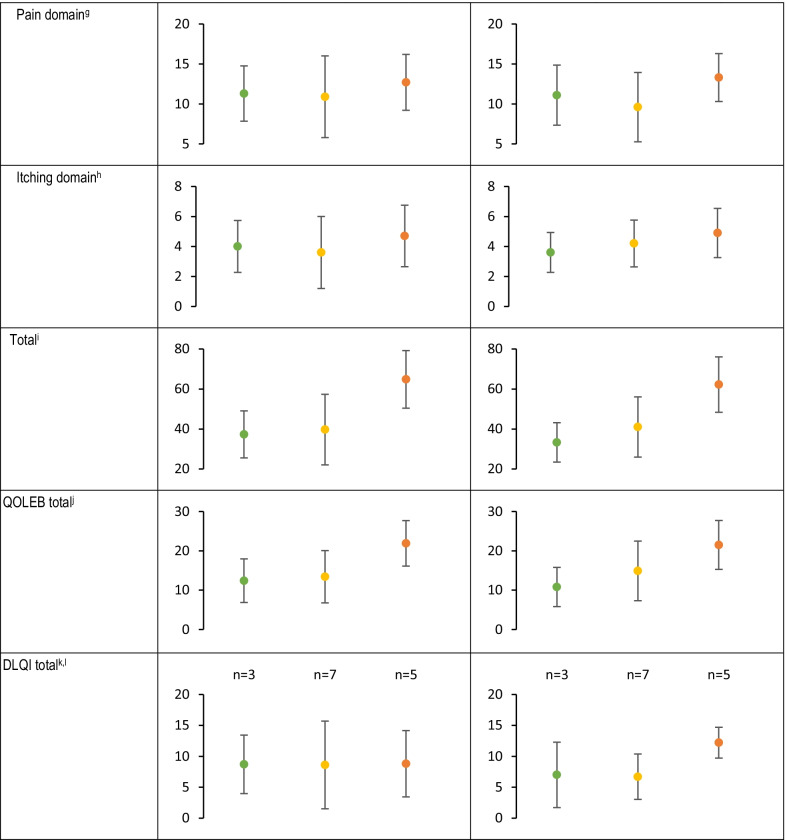

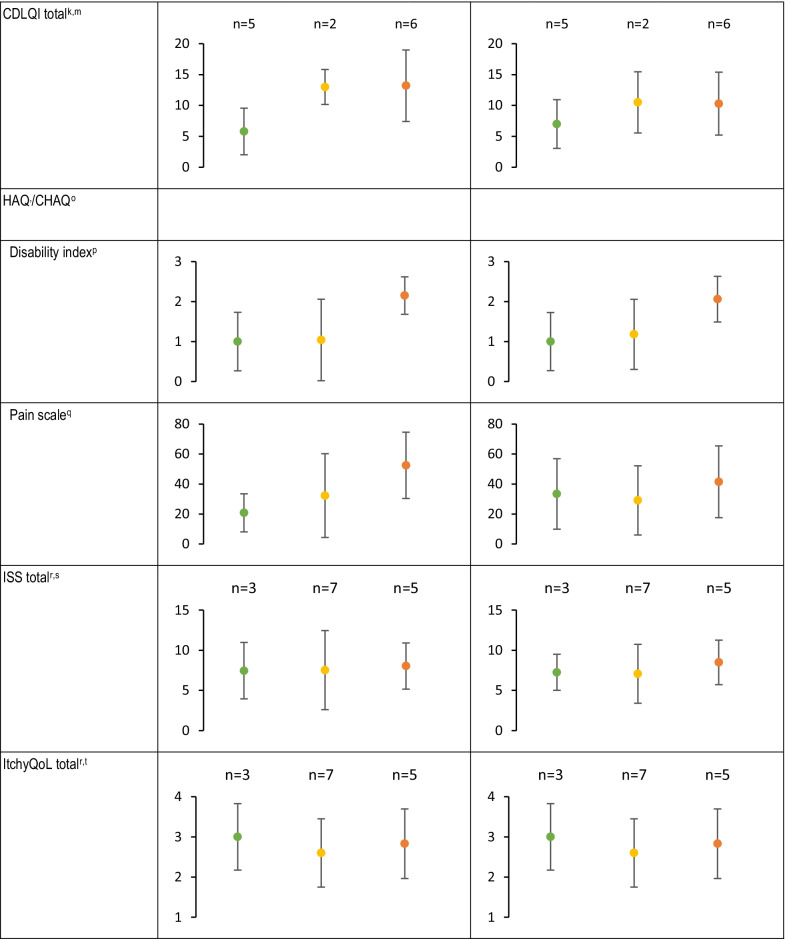
Disease-related questionnaires and instruments by disease severity

*CHAQ* Childhood HAQ, *CLDQI* Children’s DLQI, *DLQI* Dermatology Life Quality Index, *EBDASI* Epidermolysis Bullosa Disease Activity and Scarring Index, *HAQ* Health Assessment Questionnaire, *iscorEB* Instrument for Scoring Clinical Outcomes for Research of Epidermolysis Bullosa, *ISS* Itch Severity Scale, *ItchyQoL* Itching Quality of Life Survey, *QOLEB* Quality of Life in Epidermolysis Bullosa

^a^Maximum possible EBDASI activity score is 230

^b^Maximum possible EBDASI damage score is 276

^c^Maximum possible EBDASI total score is 506

^d^Maximum possible iscorEB clinician sub-scale score is 114

^e^Maximum possible iscorEB patient sub-scale score is 120

^f^Maximum skin involvement score is 60

^g^Maximum possible iscorEB pain domain score is 40

^h^Maximum possible iscorEB itching domain score is 8

^i^Maximum possible iscorEB total score is 134

^j^Maximum QOLEB total score is 51

^k^Maximum possible DLQI/CDLQI score is 30

^l^For patients > 16 years

^m^For patents 4–16 years

^n^For patients ≥ 8 years

^o^For patients < 8 years

^p^Adjusted for type of assistance usually needed; maximum possible HAQ/CHAQ disability index score is 3

^q^Maximum possible HAQ/CHAQ pain scale score is 100

^r^For patients ≥ 18 years

^s^Maximum possible ISS total score is 21

^t^Maximum possible ItchyQoL total score is 5

Minimal changes in QoL were noted from baseline to week 4 in the proportions of patients with very mild (3.3 to 3.4%, respectively), mild (16.7 to 17.2%), moderate (33.3 to 41.4%), and severe (46.7 to 37.9%), based on QOLEB. DLQI/CDLQI scores were largely unchanged during the 4-week duration of observation for patients with mild disease; scores did shift by the end of the study for patients with moderate or severe disease, but no pattern in direction of change was observed.

Overall, there was little-to-no change in HAQ/CHAQ disability index (adjusted for assistance) or pain scale, iscorEB pain domain, and itch as measured by ISS or ItchyQoL scores during the 4-week observation period (Table 4). There was no change in hand function scores during the study (Table 5).

**Table 5:** Hand function scores

Disease severity	Grade 0 (no fusion)		Grade 1 (fusion extending to the proximal interphalangeal joint)		Grade 2 (fusion extending to the distal interphalangeal joint of the longer finger)		Grade 3 (fusion extending to the tip of the digit)	
	Right hand	Left hand	Right hand	Left hand	Right hand	Left hand	Right hand	Left hand
Mild N = 9								
Baseline	9 (100)	9 (100)	0	0	0	0	0	0
Week 4	9 (100)	9 (100)	0	0	0	0	0	0
Moderate N = 10								
Baseline	5 (50.0)	5 (50.0)	3 (30.0)	3 (30.0)	0	0	2 (20.0)	2 (20.0)
Week 4	5 (50.0)	5 (50.0)	2 (20.0)	2 (20.0)	0	0	2 (20.0)	2 (20.0)
Severe N = 11								
Baseline	2 (18.2)	2 (18.2)	3 (27.3)	5 (45.5)	1 (9.1)	0	5 (45.5)	4 (36.4)
Week 4	2 (18.2)	2 (18.2)	3 (27.3)	5 (45.5)	0	0	6 (54.5)	4 (36.4)
Overall N = 30								
Baseline	16 (53.3)	16 (26.7)	6 (20.0)	8 (26.7)	1 (3.3)	0	7 (23.3)	6 (20.0)
Week 4	16 (53.3)	16 (26.7)	5 (16.7)	7 (23.3)	0	0	8 (26.7)	6 (20.0)

#### Differences across disease severities

Mean total iscorEB clinician sub-scale and skin involvement scores were numerically higher (indicating worse disease) for patients with more severe DEB, and did not substantially overlap between mild, moderate, and severe disease (Table 4). Similarly, mean EBDASI activity (overall, skin-specific, and hand and nail-specific), damage (overall, skin-specific, and hand and nail-specific), and total scores were numerically higher (indicating worse disease) for patients with more severe disease, and all except hand and nail damage did not substantially overlap between mild, moderate, and severe disease (Table 4). Mean total iscorEB patient sub-scale did not substantially overlap between patients with severe and moderate disease but did overlap between mild and moderate disease (Table 4).

Mean QOLEB and DLQI/CDLQI total scores were numerically higher (indicating worse QoL) and did not substantially overlap between patients with severe and moderate disease, but did overlap between mild and moderate disease (Table 4). Likewise, mean DLQI scores for daily activities and treatment did not substantially overlap among mild, moderate, and severe disease. Only the leisure CDLQI question did not substantially overlap between mild, moderate, and severe disease. The HAQ/CHAQ disability index (adjusted for assistance) and pain scale did not substantially overlap between moderate and severe disease, but did overlap between mild and moderate (Table 5). There was no pattern for impact of itch as assessed by ISS, ItchyQoL, or the iscorEB itching domain scores across severities (Table 4). EBDASI hand and nail damage scores increased with disease severity, with substantial overlap between groups; however, there was little difference across severities for EBDASI hand and nail activity scores (Table 4). The extent of digit fusion increased with disease severity (Table 5).

#### Age effects

Clinician-reported disease severity assessments (EBDASI activity, EBDSAI skin activity, EBDASI hand and nail activity, EBDASI damage, EBDASI skin damage, EBDASI hand and nail damage, EBDASI total, and iscorEB clinician sub-scale, iscorEB skin involvement score) were greatest for patients aged > 10 to 18 years, but there were substantial overlaps with other age groups (Additional file 4).

### Tolerability of procedures

Overall, 8 (26.7%) patients reported adverse events (AEs; 0 for mild disease, 3 [30.0%] for moderate diseases, and 5 [45.5%] for severe disease), none of which were procedure related. There was 1 (3.3%) patient with a serious AE that led to hospitalization. There were no deaths or discontinuations because of AEs.

## Discussion

In this prospective observational, 4-week study, methodologies to assess wound evolution and disease impact in patients with DEB were explored. Although mean values for total wound surface area and disease burden remained relatively stable during the 4-week observation period, individual wounds were unstable and variability among patients in all measures was quite large.

Irrespective of disease severity, healing, recurrence, and re-healing of wounds were common. This points to the potential limitation of arbitrary/indiscriminate selection of target wounds in a clinical trial as selected wounds may not be a true reflection of the efficacy of an intervention nor a comprehensive view of the overall impact of DEB. Target wounds may spontaneously heal (30% of target wounds at week 2 and 50% at week 4), and new wounds may occur (as was the case for all patients in this study). While the pattern of wound healing and recurrence was unpredictable, wounds that healed and/or healed initially and then recurred were generally smaller in size consistent with literature [18]. In addition, wounds that healed showed more signs of healing at baseline (more epithelialization, healthy granulation tissue) and lack of infection (no odor, no-to-scant exudate that was clear/not purulent) than those that persisted. Despite 30–50% of target lesions spontaneously healing, CGA of overall surface area affected by wounds and disease-related questionnaire and instrument scores were stable over the 28-day observation period. Taken together, these observations support the use of direct investigator assessment of target wounds to assess therapeutic effects in future studies, with the wounds chosen based on their chronicity and low likelihood to heal without intervention as well as global assessments of overall BSA affected and type of wounds.

This study provided insights into methods to assess the WSA of target wounds and CGA of wounds overall. The direct visualization method produced slightly higher CGA values than remote photographic assessment. The lower-than-expected estimates of WSA of selected wounds (7.9 cm^2^ overall for baseline and week 4 combined) and CGA of WSA (≤ 8.4% of BSA overall by direct visual assessment at baseline, week 2, and week 4) further suggest that additional disease severity measures may be needed to detect disease-modifying effects in a clinical trial.

This study also provided insight into the value of various disease-related questionnaires and instruments. The EBDASI, and in particular the skin-specific EBDASI sub-scales, was the only disease-related questionnaire or instrument that did not have substantial overlap between mild and moderate disease, whereas both the EBDASI and the iscorEB clinician score did not have substantial overlap between moderate and severe disease, as also reported recently by Rogers et al. [24]. In contrast, subjective measures based on patient or caregiver assessment (including the iscorEB patient sub-scale and total scores) did have substantial overlap between objective disease severities, suggesting that the extent of disease and the impact on patients are not linearly related. Taken together, a combination of clinician-assessed and patient-/caregiver-assessed measures will provide a comprehensive view of DEB severity and impact and efficacy of potential treatments in future clinical trials.

This study highlights the importance of assessing patient-/caregiver-reported outcomes scores longitudinally, as perception of symptoms and their severity may vary by patient/caregiver and be discordant from clinician-assessed outcomes. Pain, itch, and QoL measures may be particularly influenced by prior experience with pain and antipruritic medications. Patients with DEB experience pain and itch from a very early age and may have developed coping mechanisms and/or have adjusted their QoL expectations and impact of these symptoms. In addition, assessing itch and pain in clinical studies can be challenging. Both symptoms are heterogeneous in nature and can occur intermittently, making it difficult to assess trends over time. Furthermore, the itch measures included in this study were not skin-specific. In atopic dermatitis, itch and skin pain have been closely linked, particularly in children [25]. Future studies should include assessments of skin pain as well as general pain, as in the iscorEB scale.

## Limitations

A shortcoming of the study was the brevity of observation and lack of protocol-specified criteria regarding the sizes of selected wounds (patients and/or caregivers selected wounds that had the greatest impact on QoL, rather than size). Other recent EB trials were 12 weeks in duration and only assessed wounds that were ≥ 10 cm^2^ [26, 27]. The 4-week duration of this trial is helpful for the assessment of stability and precision of the included assessments but was generally not long enough to enable meaningful observation of disease progression. In addition, the small number of patients included in the study may have led to the relatively large variations in scores observed within groups and contributed to the substantial overlaps between disease severities in most of the disease-related questionnaires and instruments. Lastly, the severity classification method used in this study was loosely based on BSA, which may have been problematic. Since this study was conducted, severity strata for EBDASI (which takes into account both cutaneous and internal disease activity and damage) have been validated based on Physician’s Subjective Assessment of Severity [23].

## Conclusions

In conclusion, evaluation of disease-modifying activity for an investigational therapy for DEB will likely require direct clinical skin severity assessments that are based on carefully selected target wounds that are unlikely to spontaneously heal. Objective skin assessments should be done in combination with patient-/caregiver-reported outcomes to provide a more robust assessment of what is a truly meaningful change for patients.

## Methods

### Study design

This prospective, multicenter, multinational, observational, 4-week study (ClinicalTrials.gov, NCT02178969. Registered 4 June 2014, https://clinicaltrials.gov/ct2/show/NCT02178969) consisted of a screening period of up to 28 days; a 4-week observation period including visits at baseline, week 2, and week 4; and a safety follow-up telephone call 1 week later. Visits took place at the patient’s home, at the clinical site, or a combination of the two, depending on the investigator’s standard practice and patient or caregiver preference.

Primary outcomes were to assess wound surface area (WSA) using quantitative 3-dimensional medical imaging and clinical global assessment (CGA) of WSA as a percentage of body surface area (BSA). Secondary outcomes were to characterize changes in severity and burden of disease using patient diaries and several disease-related questionnaires, as well as to assess the tolerability of study procedures (ie, adverse event [AEs] and serious AE incidence, severity, and relationship with study procedures).

The study was conducted from June 2014 to March 2015 in accordance with GCP as described in the US FDA Title 21 Code of Federal Regulations Parts 50, 54, 56, and 312, the International Conference on Harmonisation GCP guidelines, and with the ethical principles described in the Declaration of Helsinki.

### Patients

Patients of any age with a confirmed diagnosis of DEB by genetic analysis (COL7A1 mutation) or, in the opinion of the investigator, by histologic criteria (electron microscopy or antigen mapping), ≥ 1 wound suitable for imaging, and ≥ 5 measurable wounds were eligible. Patients with current or recent locally invasive or metastatic squamous cell carcinoma or experimental intervention use were excluded. Written informed consent was obtained from patients or parents/guardians prior to participation, each approved by an institutional review board.

The study was designed to enroll patients with mild, moderate, or severe DEB based on a combination of the extent of BSA affected by wounds, DEB subtype diagnosis (by combinations of immunofluorescence mapping, electron microscopy, and/or genetic testing), body parts affected, and presence of non-cutaneous symptoms, in accordance with the international classification system [28]. Wounds were defined as blisters, erosions, or crusted erosions (ie, not with merely inflammation, dyspigmentation, or scarring) and classified as acute (duration < 3 weeks) or chronic (duration ≥ 3 weeks).

### Trial procedures

At baseline, the patient or caregiver selected 3 wounds (patient-selected wounds) that were considered to have the greatest impact on the patient’s health-related quality of life (QoL). The investigator (or designee) selected 2 additional wounds at baseline for imaging and observation throughout the study period; in cases where a wound healed at week 2, the wound area continued to be monitored through week 4 for recurrence.

Patients underwent wound-based quantitative 3-dimensional medical imaging to estimate WSA of patient- and investigator-selected selected wounds using a handheld 3-dimensional camera (Canfield Vectra H1-270) at baseline, week 2, and week 4. The images were assessed quantitatively by 3 independent, blinded central readers selected based on their knowledge of DEB and wounds. Reviewers received technical training on how to utilize central reading workstations and wound delineation software. CGA was performed at the same time points using 2 methods: direct visual assessment and remote assessment using 2-dimensional digital photographs. CGAs by both methods were calculated twice, once using the rule of nines [29] based on 12 regions and once by the PASI method [30] based on 4 regions.

Patient or caregivers assessed the patient-selected wounds weekly and recorded findings in the patient and family diary, including occurrence or recurrence of wounds.

Disease-related questionnaires and instruments were completed at baseline and week 4. Disease severity assessments comprised the EB Disease Activity and Scarring Index (EBDASI) [31] and the Instrument for Scoring Clinical Outcomes for Research of EB (iscorEB) [32]. QoL measures comprised the Quality of Life in EB Questionnaire (QOLEB) [33], the Dermatology Life Quality Index (DLQI; patients > 16 years) [34], and the Children’s DLQI (CDLQI; patients 4–16 years) [35]. Pain and disability assessments comprised the Health Assessment Questionnaire (HAQ; patients ≥ 8 years) [36], the Childhood HAQ (CHAQ; patients < 8 years) [37], and the hand function score [38, 39]. Itch assessments comprised the Itch Severity Scale (ISS; patients ≥ 18 years) [40] and the Itching QoL Survey (ItchyQoL; patients ≥ 18 years) [41]. See Additional file 5 for additional information on these disease-related questionnaires and instruments.

### Statistical analysis

Up to 30 patients with DEB were to be enrolled in the study. The sample size was not based on statistical considerations, but was chosen to assess the disease extent, impact, and wound evolution in patients for use in future studies with different levels of DEB severity (mild, moderate, and severe). At most, 10 patients with mild DEB were to be enrolled. All statistical analyses were performed using SAS^®^ software (SAS Institute Inc, Cary, NC, USA). Given the early phase and objectives of the study, the statistical methodology supporting this study focused on descriptive rather than inferential approaches. Continuous variables were summarized by means, SDs, medians, minimums, and maximums. Categorical variables were summarized by the number and proportion of patients in each category. In addition, the proportions of patients who experienced minimal clinically important differences in EBDASI (≥ 9-points reduction for improvement or ≥ 3-point increase for worsening) [23], QOLEB (≥ 6-point change) [19], and DLQI/CDLQI (DLQI, ≥ 4-point change; CDLQI, ≥ 6-point change) [20, 21] were calculated, as were the proportions of patients with mild (total score 0–42), moderate (total score 43–106), and severe (total score 107–506) DEB based on EBDASI [23]; very mild (score of 0–4), mild (5–9), moderate (10–19), severe (20–34), or very severe (35–51) impairment in QoL based on QOLEB [19]; and no effect (DLQI/CDLQI score of 0–1), small effect (DLQI, 2–5; CDLQI, 2–6), moderate effect (DLQI, 6–10; CDLQI, 7–12), very large effect (DLQI, 11–20; CDLQI, 13–18), or extremely large effect (DLQI, 21–30; CDLQI, 19–30) on QoL based on DLQI/CDLQI [20, 21].

## Supplementary Information

**Additional file 1.** Investigators and central readers. List of investigators and central readers who participated in this study.

**Additional file 2.** Baseline characteristics of wounds that were healed, recurrent, or still present at week 2 and week 4. Lists baseline wound characteristics by wound status at week 2 and week 4.

**Additional file 3.** Distribution of impact on quality of life by skin involvement. Lists distribution of impact on quality of life as assessed by QOLEB, DLQI, and CDLQI by skin involvement.

**Additional file 4.** Disease-related questionnaires and instruments by age group. Shows distribution of disease-related questionnaire and instrument scores by age group.

**Additional file 5.** Additional methods. Includes additional information on scales used in this study.

## Data Availability

All available data generated during this study are included in this published article and its additional files.

## Abbreviations

AE: Adverse event
BSA: Body surface area
CDLQI: Children’s Dermatology Life Quality Index
CGA: Clinical global assessment
CHAQ: Childhood Health Assessment Questionnaire
DEB: Dystrophic epidermolysis bullosa
DLQI: Dermatology Life Quality Index
EB: Epidermolysis bullosa
EBDASI: Epidermolysis Bullosa Disease Activity and Scarring Index
HAQ: Health Assessment Questionnaire
iscorEB: Instrument for Scoring Clinical Outcomes for Research of Epidermolysis Bullosa
ISS: Itch Severity Scale
ItchyQoL: Itching Quality of Life Survey
QoL: Quality of life
QOLEB: Quality of Life in Epidermolysis Bullosa
WSA: Wound surface area
