# Supplementary material for: A prospective short-term study to evaluate methodologies for the assessment of disease extent, impact, and wound evolution in patients with dystrophic epidermolysis bullosa
Source: Orphanet J Rare Dis. 2022 Aug 13;17:314. doi: 10.1186/s13023-022-02461-z (PMC9375287; doi:10.1186/s13023-022-02461-z)
Supplement: Supplementary file 1 — Additional file 1. Investigators and central readers. List of investigators and central readers who participated in this study. [file 13023_2022_2461_MOESM1_ESM.pdf]

ADDITIONAL FILE 1

Investigators and Central Readers

**Investigator**	**Affiliation**
Dedee F Murrell, MD	The St George Hospital Clinical School, University of New South Wales and Premier Specialists & Dermatology Trials Australia, Sydney, Australia
Johann Bauer, MD, MBA	Paracelsus Medical University Salzburg, EB House Austria, University Hospital of Dermatology and Mullner Hauptstraße, Salzburg, Austria
Hana Buckova, MD, PhD	Fakultní nemocnice Brno, Dětská nemocnice, Kožní oddělení Pediatrické kliniky, Brno, Czech Republic
Alain Hovnanian, MD, PhD	Necker Hospital for Sick Children and University Paris Descartes, Paris, France
Johannes Kern, MD, PhD	University Medical Center Freiburg, Freiburg, Germany
May El Hachem, MD	Ospedale Pediatrico Bambino Gesù, Rome, Italy
Giovanna Zambruno, MD<br>Liliana Guerra, MD, PhD	Laboratory of Molecular and Cell Biology, lstituto Dermopatico dell'lmmacolata, Rome, Italy
Richard Azizkhan, MD	Cincinnati Children’s Hospital Medical Center, Cincinnati, Ohio, USA
Amy S Paller, MD, MS	The Feinberg School of Medicine of Northwestern University, Chicago, Illinois, USA
**Central Reader**	**Affiliation**
Irene Lara-Corrales, MD	The Hospital for Sick Children, Toronto, ON, Canada
Afsaneh Alavi, MSc, MD	Women College Hospital, Toronto, ON, Canada
Cezary Kowalewski, MD	Medical University of Warsaw, Warsaw, Poland
