# Supplementary material for: A prospective short-term study to evaluate methodologies for the assessment of disease extent, impact, and wound evolution in patients with dystrophic epidermolysis bullosa
Source: Orphanet J Rare Dis. 2022 Aug 13;17:314. doi: 10.1186/s13023-022-02461-z (PMC9375287; doi:10.1186/s13023-022-02461-z)
Supplement: Supplementary file 2 — Additional file 2. Baseline characteristics of wounds that were healed, recurrent, or still present at week 2 and week 4. Lists baseline wound characteristics by wound status at week 2 and week 4. [file 13023_2022_2461_MOESM2_ESM.pdf]

ADDITIONAL FILE 2

Baseline Characteristics of Wounds that Were Healed, Recurrent, or Still Present at Week 2 and Week 4

		Healed		Recurred		Still Present	
		Week 2 n=45	Week 4 n=75	Week 2 n=8	Week 4 n=14	Week 2 n=87	Week 4 n=56
**Wounds, n (%)**							
Epithelization	None	9 (20.0)	15 (20.0)	4 (50.0)	3 (21.4)	12 (13.8)	7 (12.5)
	<25%	11 (24.4)	24 (32.0)	0	6 (42.9)	45 (51.7)	26 (46.4)
	25%–49%	4 (8.9)	7 (9.3)	1 (12.5)	1 (7.1)	11 (12.6)	8 (14.3)
	50%–74%	8 (17.8)	15 (20.0)	0	1 (7.1)	10 (11.5)	5 (8.9)
	75%–100%	10 (22.2)	13 (17.3)	3 (37.5)	1 (7.1)	7 (8.0)	8 (14.3)
	Other	3 (6.7)	1 (1.3)	0	2 (14.3)	2 (2.3)	2 (3.6)
Granulation tissue	No	10 (22.2)	26 (34.7)	4 (50.0)	2 (14.3)	17 (19.5)	8 (14.3)
	Yes, healthy	33 (73.3)	48 (64.0)	3 (37.5)	11 (78.6)	59 (67.8)	36 (64.3)
	Yes, hypergranulation	2 (4.4)	1 (1.3)	0	1 (7.1)	8 (9.2)	8 (14.3)
	Yes, other	0	0	1 (12.5)	0	3 (3.4)	4 (7.1)
Wound appearance	Clean	29 (64.4)	49 (65.3)	5 (62.5)	8 (57.1)	55 (63.2)	36 (64.3)
	Not clean	16 (35.6)	26 (34.7)	3 (37.5)	6 (42.9)	32 (36.8)	20 (35.7)
Exudate type	Not purulent	44 (97.8)	71 (94.7)	6 (75.0)	14 (100)	75 (86.2)	45 (80.4)
	Purulent	1 (2.2)	4 (5.3)	2 (25.0)	0	12 (13.8)	11 (19.6)
Exudate color	Clear	20 (44.4)	31 (41.3)	1 (12.5)	5 (35.7)	25 (28.7)	11 (19.6)
	Not clear	25 (55.6)	44 (58.7)	7 (87.5)	9 (64.3)	62 (71.3)	45 (80.4)
Exudate amount	None	18 (40.0)	23 (30.7)	5 (62.5)	4 (28.6)	13 (14.9)	9 (16.1)
	Scant (>25% dressing soiled)	19 (42.2)	36 (48.0)	2 (25.0)	4 (28.6)	34 (39.1)	18 (32.1)
	Mild (25%– 49% dressing soiled)	7 (15.6)	13 (17.3)	1 (12.5)	4 (28.6)	22 (25.3)	5 (26.8)
	Moderate (50%–75% dressing soiled)	0	2 (2.7)	0	1 (7.1)	15 (17.2)	12 (21.4)
	Heavy (>75% dressing soiled/strikethrough)	1 (2.2)	1 (1.3)	0	1 (7.1)	3 (3.4)	2 (3.6)
Wound odor	No	43 (95.6)	67 (89.3)	6 (75.0)	14 (100)	65 (74.7)	38 (67.9)
	Yes	2 (4.4)	7 (9.3)	2 (25.0)	0	21 (24.1)	18 (32.1)
Wound margins definition	Well defined	32 (71.1)	53 (70.7)	4 (50.0)	12 (85.7)	66 (75.9)	40 (71.4)
	Poorly defined	13 (28.9)	22 (29.3)	4 (50.0)	2 (14.3)	21 (24.1)	16 (28.6)
Wound appearance	Healthy	27 (60.0)	47 (62.7)	6 (75.0)	9 (64.3)	57 (65.5)	39 (69.6)
	Unhealthy, active	10 (22.2)	15 (20.0)	2 (25.0)	2 (14.3)	15 (17.2)	10 (17.9)
	Unhealthy, hyperkeratotic/fibrotic	1 (2.2)	2 (2.7)	0	0	2 (2.3)	1 (1.8)
	Unhealthy, active and hyperkeratotic/fibrotic	7 (15.6)	10 (13.3)	0	3 (21.4)	11 (12.6)	5 (8.9)
	Unhealthy, other	0	1 (1.3)	0	0	2 (2.3)	1 (1.8)
Peri-wound tissue	Normal	6 (13.3)	8 (10.7)	2 (25.0)	0	13 (14.9)	13 (23.2)
	Abnormal, red	32 (71.1)	46 (61.3)	3 (37.5)	9 (64.3)	46 (52.9)	26 (46.4)
	Abnormal, not red	7 (15.6)	21 (28.0)	3 (37.5)	5 (35.7)	28 (32.2)	17 (30.4)

		Healed		Recurred		Still Present	
		Week 2 n=45	Week 4 n=75	Week 2 n=8	Week 4 n=14	Week 2 n=87	Week 4 n=56
Wound depth	Shallow	38 (84.4)	63 (84.0)	8 (100)	10 (71.4)	70 (80.5)	48 (85.7)
	Medium	6 (13.3)	10 (13.3)	0	3 (21.4)	14 (16.1)	7 (12.5)
	Deep	0	0	0	1 (7.1)	1 (1.1)	0
Scarring	No	19 (42.2)	27 (36.0)	1 (12.5)	2 (14.3)	29 (33.3)	20 (35.7)
	Yes, hypertrophic	2 (4.4)	2 (2.7)	0	3 (21.4)	8 (9.2)	5 (8.9)
	Yes, atrophic	19 (42.2)	35 (46.7)	5 (62.5)	7 (50.0)	38 (43.7)	25 (44.6)
	Yes, other	5 (11.1)	11 (14.7)	2 (25.0)	2 (14.3)	12 (13.8)	6 (10.7)
Wound infection	No	44 (97.8)	70 (93.3)	6 (75.0)	14 (100)	76 (87.4)	47 (83.9)
	Yes	1 (2.2)	5 (6.7)	2 (25.0)	0	11 (12.6)	9 (16.1)
Score, mean	Itch	3.5	3.5	8.3	4.6	3.6	4.3
	Pain	3.2	3.3	6.6	3.9	4.2	4.9
	Sensitivity	4.0	4.1	6.6	5.1	5.0	5.7
	Sensation	3.3	3.1	5.9	4.2	3.6	3.9
