# Supplementary material for: A prospective short-term study to evaluate methodologies for the assessment of disease extent, impact, and wound evolution in patients with dystrophic epidermolysis bullosa
Source: Orphanet J Rare Dis. 2022 Aug 13;17:314. doi: 10.1186/s13023-022-02461-z (PMC9375287; doi:10.1186/s13023-022-02461-z)
Supplement: Supplementary file 3 — Additional file 3. Distribution of impact on quality of life by skin involvement. Lists distribution of impact on quality of life as assessed by QOLEB, DLQI, and CDLQI by skin involvement. [file 13023_2022_2461_MOESM3_ESM.pdf]

ADDITIONAL FILE 3

Distribution of Impact on Quality of Life by Disease Severity

	Patients, n (%)							
	Mild		Moderate		Severe		Overall	
	BL	Wk4	BL	Wk4	BL	Wk4	BL	Wk4
**QOLEB**	**N=9**	**N=9**	**N=10**	**N=9**	**N=11**	**N=11**	**N=30**	**N=29**
Very mild (0-4)	1 (11.1)	1 (11.1)	0	0	0	0	1 (3.3)	1 (3.4)
Mild (5-9)	1 (11.1)	2 (22.2)	4 (40.0)	3 (33.3)	0	0	5 (16.7)	5 (17.2)
Moderate (10-19)	6 (66.7)	5 (55.6)	2 (20.0)	3 (33.3)	2 (18.2)	4 (36.4)	10 (33.3)	12 (41.4)
Severe (20-34)	1 (11.1)	1 (11.1)	4 (40.0)	3 (33.3)	9 (81.8)	7 (63.6)	14 (46.7)	11 (37.9)
**DLQI**	**N=3**	**N=3**	**N=7**	**N=6**	**N=5**	**N=5**	**N=15**	**N=14**
No effect (0-1)	0	1 (33.3)	0	0	0	0	0	1 (6.7)
Small effect (2-5)	1 (33.3)	0	4 (57.1)	3 (42.9)	1 (20.0)	0	6 (40.0)	3 (20.0)
Moderate effect (6-10)	1 (33.3)	1 (33.3)	1 (14.3)	2 (28.6)	2 (40.0)	2 (40.0)	4 (26.7)	5 (33.3)
Very large effect (11-20)	1 (33.3)	1 (33.3)	1 (14.3)	1 (14.3)	2 (40.0)	3 (60.0)	4 (26.7)	5 (33.3)
Extremely large effect (21-30)	0	0	1 (14.3)	0	0	0	1 (6.7)	0
**CDLQI**	**N=5**	**N=5**	**N=2**	**N=2**	**N=6**	**N=6**	**N=13**	**N=13**
No effect (0-1)	0	0	0	0	0	0	0	0
Small effect (2-6)	4 (80.0)	2 (40.0)	0	0	1 (16.7)	1 (16.7)	5 (38.5)	3 (23.1)
Moderate effect (7-12)	1 (20.0)	2 (40.0)	1 (50.0)	1 (50.0)	1 (16.7)	4 (66.7)	3 (23.1)	7 (53.8)
Very large effect (13-18)	0	1 (20.0)	1 (50.0)	1 (50.0)	3 (50.0)	0	4 (30.8)	2 (15.4)
Extremely large effect (19-30)	0	0	0	0	1 (16.7)	1 (16.7)	1 (7.7)	1 (7.7)

DLQI was completed by patients >16 years. CDLQI was completed by patients 4-16 years.
BL, baseline; CLDQI, Children’s DLQI; DLQI, Dermatology Life Quality Index; QOLEB, Quality of Life in Epidermolysis Bullosa; wk, week.
