# Supplementary material for: A prospective short-term study to evaluate methodologies for the assessment of disease extent, impact, and wound evolution in patients with dystrophic epidermolysis bullosa
Source: Orphanet J Rare Dis. 2022 Aug 13;17:314. doi: 10.1186/s13023-022-02461-z (PMC9375287; doi:10.1186/s13023-022-02461-z)
Supplement: Supplementary file 4 — Additional file 4. Disease-related questionnaires and instruments by age group. Shows distribution of disease-related questionnaire and instrument scores by age group. [file 13023_2022_2461_MOESM4_ESM.pdf]

ADDITIONAL FILE 4

Disease-Related Questionnaires and Instruments by Age Group

Mean (SD)	Baseline	Week 4
	>2 to 10 ye ars N=10 · >10 to 18 y ears N=5 · >18 year s N=15	>2 to 10 ye ars N=10 · >10 to 18 y ears N=5 · >18 year s N=15
**EBDASI**		
Activity[a]	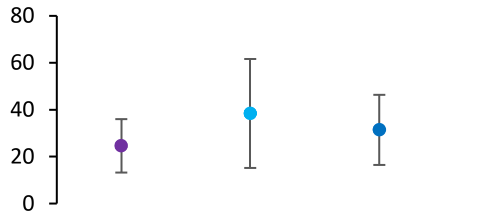	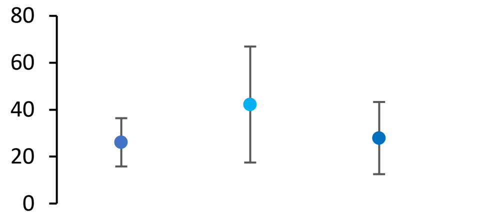
Skin activity	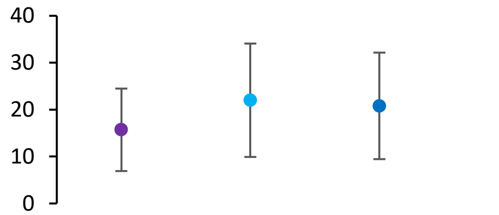	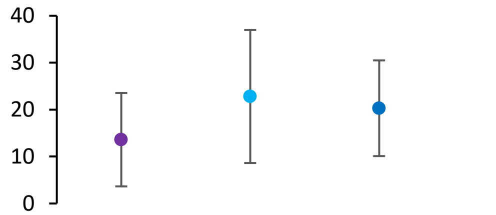
Hand and nail activity	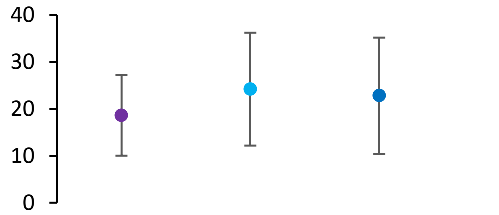	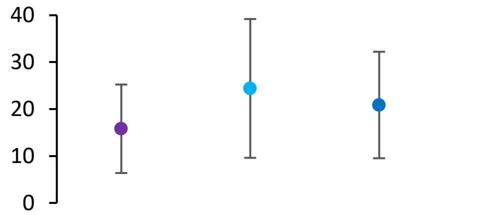
Damage[b]	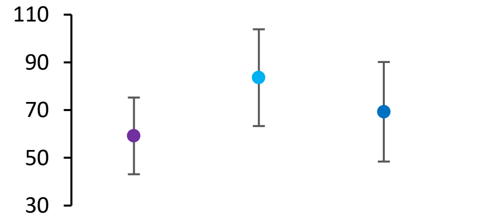	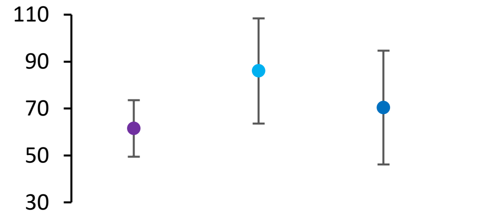
Skin damage	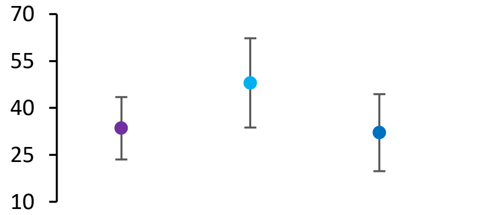	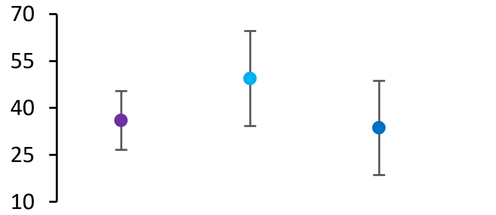

Mean (SD)	Baseline<br>>2 to 10 ye ars N=10 · >10 to 18 y ears N=5 · >18 year s N=15	Week 4<br>>2 to 10 ye ars N=10 · >10 to 18 y ears N=5 · >18 year s N=15
Hand and nail damage	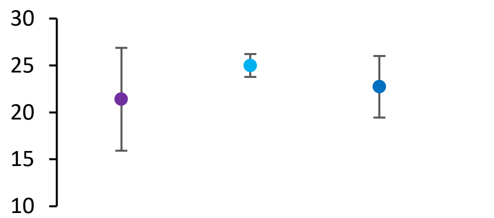	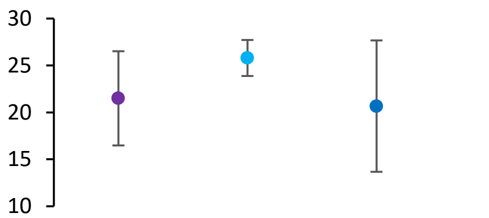
Total[c]	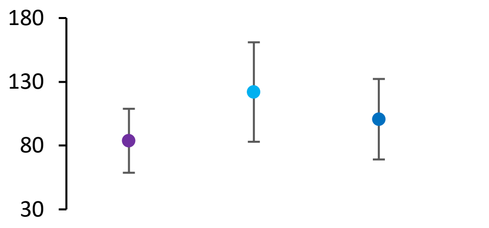	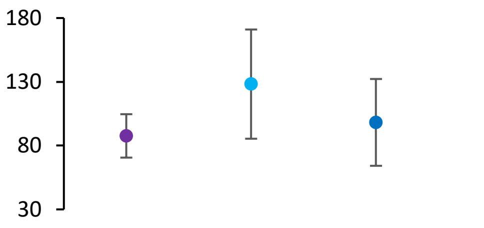
**iscorEB**		
Clinician sub-scale[d]	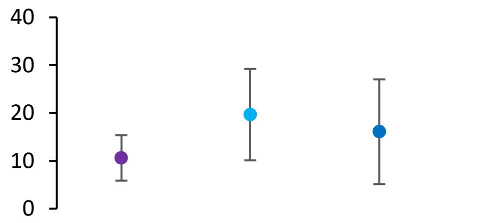	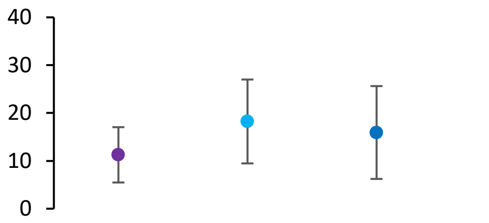
Patient sub-scale[e]	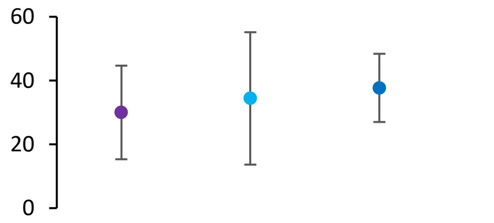	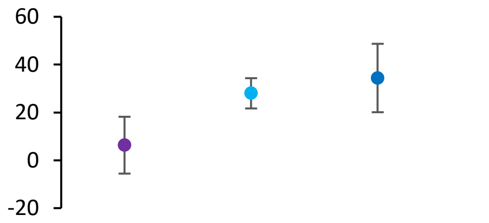
Skin involvement score[f]	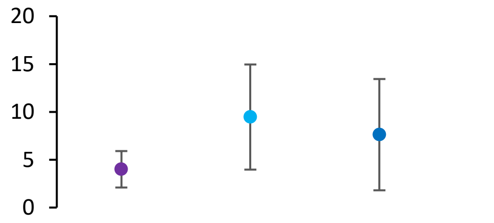	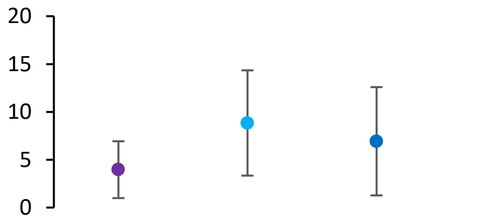

[a]Maximum possible EBDASI activity score is 230.
[b]Maximum possible EBDASI damage score is 276.
[c]Maximum possible EBDASI total score is 506.
[d]Maximum possible iscorEB clinician sub-scale score is 114.
[e]Maximum possible iscorEB patient sub-scale score is 120.
[f]Maximum possible iscorEB skin involvement score is 60.

EBDASI, Epidermolysis Bullosa Disease Activity and Scarring Index; iscorEB, Instrument for Scoring Clinical Outcomes for Research of Epidermolysis Bullosa.
