# Supplementary material for: A prospective short-term study to evaluate methodologies for the assessment of disease extent, impact, and wound evolution in patients with dystrophic epidermolysis bullosa
Source: Orphanet J Rare Dis. 2022 Aug 13;17:314. doi: 10.1186/s13023-022-02461-z (PMC9375287; doi:10.1186/s13023-022-02461-z)
Supplement: Supplementary file 5 — Additional file 5. Additional methods. Includes additional information on scales used in this study. [file 13023_2022_2461_MOESM5_ESM.pdf]

ADDITIONAL FILE 5

Supplementary Methods

The EBDASI [1] and iscorEB [2] were developed for patients with EB, though not specifically for the DEB subtype. The EBDASI consists of clinician-reported assessments of activity and damage. The EBDASI activity score is the sum of activity scores (from 0 [absent] to 10 [entire area involved]) for 12 skin areas (ears, face, neck, chest, abdomen, back, buttocks, arms, hands, legs, feet, anogenital), scalp, mucous membranes (eyes, nose, buccal mucosa, hard palate, soft palate, upper gingiva, lower gingiva, tongue, floor of mouth, labial mucosa, posterior pharynx, anogenital), and nails (fingernails and toenails) and activity (scored as 0 [absent] or 2 [present]) for other epithelialized surfaces (larynx, esophagus, genitourinary) for a maximum possible activity score of 230. The EBDASI damage score is the sum of damage scores for skin (erythema, dyspigmentation, poikiloderma, skin atrophy, hyperkeratosis/scaling, scarring, milia each scored as 0 [absent] or 1 [present] out of 7 for the above 12 skin areas), scalp (hyperpigmentation/hypopigmentation/hyperkeratosis, scarring alopecia each scored from 0 [absent] to 10 [entire area involved]), mucous membranes (ectropion, symblepharon, visible cornea opacity, clinical microsomia, ankyloglossia, intraoral scars, enamel hypoplasia, anal structures each scored as 0 [absent] or 2 [present]), nails (number of dystrophic nails plus 3 points for each anonychia), and other epithelialized surfaces (larynx, esophagus, genitourinary, hands, skin cancer each scored from 0 [none] to 10 [worst damage]) for a maximum possible damage score of 276. Activity and damage scores are summed for a maximum EBDASI total score of 506.

The iscorEB consists of both clinician-reported and patient-/caregiver-reported outcomes. Clinicians assessed current skin involvement (intact blisters, erosions, crusting/scabbing, chronic wounds, infection, and BSA involved for the head/neck, upper extremities, trunk, and lower extremities); mucosal involvement in previous 4 weeks (eg, mouth erosions, stridor, hoarseness, eye redness, palpebral closure); internal organ involvement in previous 6 months (body mass index, tube feeding, renal disease, decreased cardiac function); laboratory abnormalities in previous 6 months (anemia, low albumin, inflammation);

and complications/procedures in previous 12 months (squamous cell carcinomas, osteopenia/osteoporosis, hospital visits, esophageal dilatation) for a maximum clinician sub-scale score of 114 points. Patients or caregivers assessed pain (overall, skin, mouth, eye, bone/joint), itching, essential functions (eating/drinking, having bowel movements, urinating/voiding), sleeping, daily activities (moving around, using hands), mood, and disease impact (leisurely activities, work/school/learning) over the previous 4 weeks each rated from 0 (none) to 8 (worst possible/unable to) for a maximum patient sub-scale score of 120 points. Clinician and patient sub-scale scores are summed to yield a total iscorEB score with a maximum possible score of 234.

QOLEB [3] is a 17-item QoL instrument developed and validated for patients with various types of EB. The maximum QOLEB score of 51 indicates worst QoL. DLQI [4] and CDLQI [5] are 10-item dermatology-specific QoL instruments with maximum possible scores of 30 indicating greatest impact on QoL.

The HAQ [6] and CHAQ [7] are generic questionnaires that quantify disability (average of 8 disability components [dressing and grooming, arising, eating, walking, hygiene, reach, grip, and activities] each rated from 0 [without any difficulty] to 3 [unable to do]) and pain (rated on a visual analog scale from 0 [no pain] to 100 [severe pain]). HAQ/CHAQ disability index scores of 0–1 are generally considered to represent mild to moderate difficulty, 1–2 moderate-to-severe disability, and 2–3 severe-to-very severe disability.

The hand function assessment [8, 9] is a rating scale of simple syndactyly rated as Grade 0 (no fusion), Grade 1 (fusion extending to the proximal interphalangeal joint), Grade 2 (fusion extending to the distal interphalangeal joint of the longer finger), or Grade 3 (fusion extending to the tip of the digit) for each hand.

The ISS [10] is a 7-item questionnaire used to quantify the frequency, location, intensity, sensation, and impact of pruritus with an unspecified recall period, with a maximum score of 21. The ItchyQoL [11]

total score is the average of responses to 22 items that quantifies the symptoms and functional and emotional impact of pruritus during the previous 7 days, with a maximum score of 5.

REFERENCES

1. Loh CC, Kim J, Su JC, Daniel BS, Venugopal SS, Rhodes LM, et al. Development, reliability, and validity of a novel Epidermolysis Bullosa Disease Activity and Scarring Index (EBDASI). J Am Acad Dermatol. 2014;70(1):89-97.e1-13.

2. Bruckner AL, Fairclough DL, Feinstein JA, Lara-Corrales I, Lucky AW, Tolar J, et al. Reliability and validity of the instrument for scoring clinical outcomes of research for epidermolysis bullosa (iscorEB). Br J Dermatol. 2018;178(5):1128-34.

3. Frew JW, Martin LK, Nijsten T, Murrell DF. Quality of life evaluation in epidermolysis bullosa (EB) through the development of the QOLEB questionnaire: an EB-specific quality of life instrument. Br J Dermatol. 2009;161(6):1323-30.

4. Finlay AY, Khan GK. Dermatology Life Quality Index (DLQI)--a simple practical measure for routine clinical use. Clin Exp Dermatol. 1994;19(3):210-6.

5. Lewis-Jones MS, Finlay AY. The Children's Dermatology Life Quality Index (CDLQI): initial validation and practical use. Br J Dermatol. 1995;132(6):942-9.

6. Bruce B, Fries JF. The Stanford Health Assessment Questionnaire: a review of its history, issues, progress, and documentation. J Rheumatol. 2003;30(1):167-78.

7. Fries JF, Spitz P, Kraines RG, Holman HR. Measurement of patient outcome in arthritis. Arthritis Rheum. 1980;23(2):137-45.

8. Terrill PJ, Mayou BJ, Pemberton J. Experience in the surgical management of the hand in dystrophic epidermolysis bullosa. Br J Plast Surg. 1992;45(6):435-42.

9. Colville J. Syndactyly correction. Br J Plast Surg. 1989;42(1):12-6.

10. Majeski CJ, Johnson JA, Davison SN, Lauzon CJ. Itch Severity Scale: a self-report instrument for the measurement of pruritus severity. Br J Dermatol. 2007;156(4):667-73.

11. Desai NS, Poindexter GB, Monthrope YM, Bendeck SE, Swerlick RA, Chen SC. A pilot quality-of-life instrument for pruritus. J Am Acad Dermatol. 2008;59(2):234-44.
